# 

**Published:** 1909-11-13

**Authors:** 


### BOOKS RECEIVED.

Kegan Paul, Trench, Trübner and Co., Ltd.
"The Periodic Law." By A. E. Garret, B.Sc.

Baillière, Tindall, and Cox.
"The Morphia Habit." By T. Jennings, M.D.
"The Sanatorium Treatment of Pulmonary Tuberculosis." By F. Rufenacht Walters, M.D., B.S., M.R.C.P., F.R.C.S.
"Clinical Memoranda." By A. Theodore Brand, M.D., C.M., and John Robert Keith, M.D., C.M.

A. Constable and Co.
"Studies in Tuberculosis." By Henry Clarke, M.A., M.D.

Edward Arnold.
"A System of Clinical Medicine." 2nd edition. By Thos. Dixon Savill, M.D., etc.

Sampson, Low, Marston and Co., Ltd.
"From the Four Winds." By Francis Sinclair.

### APPOINTMENTS.

Dr. Henry Joseph Cates, a member of the health staff of the Port of London Sanitary Authority, has been appointed assistant medical officer of health of Coventry.

Dr. A. M. H. Gray, M.R.C.P., has been appointed physician in charge of the skin department of University College Hospital, London.

The Hebdomadal Council have elected Mr. A. P. Dodds-Parker, B.M., M.A., Magdalen College, to be Litchfield Clinical Lecturer in Surgery for two years from January 5 next.

Mr. J. P. Bibby, M.B., Ch.B. (Leeds) has been appointed house physician, and Mr. J. D. Benjafield, M.R.C.S., L.R.C.P. (University College Hospital), house surgeon to the Evelina Hospital for Sick Children.

The Council of the University of Sheffield has made the following appointments : Mr. George Wilkinson, B.A., M.B., F.R.C.S., to the newly instituted Lectureship in Diseases of the Ear, Nose, and Throat. Mr. George H. Pooley, B.A., F.R.C.S. (Eng.), F.R.C.S. (Edin.), to the Lectureship in Ophthalmology.

### APPOINTMENTS VACANT.

Leicester Infirmary.—Assistant house physician.

West Suffolk Education Committee.—Medical inspector of school children.

### GIFTS AND BEQUESTS.

A legacy of £5,000 was bequeathed to St. Bartholomew's Hospital by the late Mrs. Annie Jane Holborn.

The Governor and Court of the Bank of England have voted £1,000 to the special fund for St. Bartholomew's Hospital in response to an appeal by the Lord Mayor. An anonymous donor has contributed £250.

The secretary of the Kensington and Fulham General Hospital has received from the proprietors of the Kensington Picture Theatre, 175 High Street, their receipts for Thursday, October 7, which day was set apart by them for the hospital's benefit.

The committee for the removal of King's College Hospital to South London has received a cheque for £100 as from the late Dr. Crawford Hayes, making up the sum of £200 contributed to the fund by Dr. and Mrs. Hayes.

A sum of over £61,000 has been bequeathed to Glasgow and West of Scotland institutions by the late Mr. Alexander Fleming, of Messrs. William Baird and Co., coalowners. Under his will he allocates £10,000 to the Glasgow Western Infirmary.

Mr. George Hadfield, of Hollywood, Pendleton, one of the founders of the Northern Counties Hospital, left estate of the gross value of £215,044 14s. 1d., and the net of £140,772 17s. 3d.

The following among other annual subscriptions have been received for King Edward's Hospital Fund for London :—Army and Navy Co-operative Society, Limited, £105; Mr. Francis Reckitt, £52 10s.; "W.," £50; Mr. Frederick M. Fry, £25; Mrs. Henry Hudson, £25; Messrs. Macmillan and Co., Limited, £25; Goldsmiths and Silversmiths Company, Limited, £21; and a donation of £100 from the Hon. F. H. Baring.

Two hundred preference shares in W. F. Stanley and Company, Limited, scientific instrument makers, have been left to the Croydon General Hospital, subject to prior life interests of relatives, by the late W. F. Stanley, J.P., and one hundred similar shares to the British Home and Hospital for Incurables at Streatham.

The late Mr. Patrick McGuinness, of Belfast, has left £500 to the Mater Infirmorum Hospital, Belfast.

The late Mrs. Elizabeth Pennell, of Dublin, has left £50 to the Hospital for Incurables, Donnybrook.

The estate of the late Dr. Hubert Elwyn Jones Biss, M.A., M.D., of Eastbourne, has been valued at £3,731 18s. 11d. gross and £2,829 11s. 5d. net.

The Rev. Frederick Edward Tyrwhitt Drake, has left £20 to the Yeatfield Hospital, Dorchester.

Dr. Edward Clapton, formerly physician and lecturer at St. Thomas's Hospital, bequeathed £50 to the Stamford and Rutland Infirmary.

The Salters' Company has sent a subscription of £10 10s. to the After-Care Association for Poor Persons discharged recovered from Asylums for the Insane, Church House, Westminster.

Mrs. Clara Maria Wilson, of Tunbridge Wells, left £600 to the Children's Hospital, Sheffield, to endow a cot.

Mr. James Sampson, of Sheffield, left £50 each to the Sheffield Public Hospital and Dispensary, and the Sheffield General Infirmary.

The Rev. Warneford Seymour Trinmere Gompertz, M.A., of Ventnor, left conditional bequests of £1,200 to the Brompton Hospital for Consumption, and £1,050 to the Royal National Hospital for Consumption, Ventnor, to increase the endowment fund of that institution.

### THE QUESTION BOX.

**SPECIAL NOTICE.**—Questions of interest affecting the honorary medical staff and hospital administration in all departments are invited. When any point arises we hope our readers will formulate it in a question and so co-operate to make this column of real value and interest. In this way the experience of the best-managed hospitals should be made helpful to the whole hospital world. We shall welcome the contribution of both questions and answers.

**N.B.**—The publication of an answer in this column does not necessarily preclude the publication of subsequent answers to the same question, for a question may involve points which require to be threshed out and fully discussed. Answers, if published, will be paid for at scale rates.

### THE OPERATION THEATRE.

Question 1. What hospitals in the provinces have the most approved and up-to-date theatres?

Note.—The Secretary-Superintendent of a hospital wishing to erect a new theatre desires this information. Will some of our correspondents answer this question, with plans and particulars of the material used for the floor of the theatre?

### HOSPITAL FLOORS AND LINOLEUM.

Question 2. What are the objections to the use of linoleum, and what is the best floor for hospital use?

The Case for Jointless Flooring.

Mr. H. Wilson Young, M.I.E.E., A.M.I.M.E., writes :

In your issue of November 6, page 167, under the heading of "Some Continental Hospitals' Practice," a statement is made respecting jointless flooring which I hope you will extend to me the courtesy of space to refute. Mr. W. Milburn, Jun., with these words, "A good many so-called jointless floors largely used in Germany a number of years ago on their invention, are now practically discarded owing to their unreliability," dismisses an industry which has assumed on the Continent, and in Germany in particular, very large proportions, and which in England during the past ten years has been steadily gaining ground.

On behalf of the jointless flooring industry of this country, I take it upon myself to most emphatically repudiate the above statement. Unreliability? In what respects? Is it on account of cracking or crumbling, non-durability, or loss of hygienic properties through absorption, or what? It may interest your correspondent to know that, owing to the great strides made in jointless flooring in Germany, the leading manufacturers founded, now some considerable time ago, a laboratory, placing at the head an eminent chemist, Dr. Donath, for the purpose of conducting research work in this particular industry with a view of arriving at standard mixtures suitable for various kinds of subfloors met with in building construction, and generally scientifically assist in the advancement of this industry. It is at least reasonable to suppose that the expense of maintaining such an institution would not be incurred by German manufacturers —who are counted in commercial circles as representing "thoroughness and method" second to none—unless the status and prospects of the business warranted such expenditure. Again, this "so-called jointless flooring, etc., practically discarded" statement prompts me to say that only three months ago in Leipzig the largest Building Trades' Exhibition ever held in Germany (corresponding to our Olympia Building Trades' Exhibition in London) gave jointless flooring two special sections to itself—one for the exhibition of finished floors and raw materials used in their construction, the other displaying works, plants, portable mixing sets, tools, etc., in which branch of mechanical engineering Germany has made a special study owing to the urgent demand for such plant and tools brought about by the great increase of this industry. Furthermore, one of the leading weekly organs of the German building trades, the *Baumaterialien-Markt*, devotes special pages to the jointless-flooring industry and recounts the weekly deliberations of the Union of Jointless Flooring Manufacturers—a most representative body comprising the heads of the leading jointless-flooring firms in Germany.

With regard to the merits of jointless flooring *versus* linoleum for hospital work in particular, it is admitted by competent and experienced authorities, both in medical administration and in building construction, that linoleum leaves much to be desired both from a durability point of view and, more particularly, from hygienic aspects. Jointless floor-laying is an art which time and experience have developed, and your correspondent must be speaking from information acquired on the results of the class of work done in the earliest stages. Reliable and durable jointless floors cannot be laid at the price of even a good-quality linoleum; but first cost, when the merits of both materials are competently weighed, is not the greatest consideration. With regard to the amount of jointless-flooring work now being done in England, I am only able to speak of what my firm has done; but as this letter is not intended as a trade advertisement, I will not seek to take up further space in your valuable paper by giving a list of hospitals in which we have laid jointless flooring, but may state that the number in London alone would cause considerable surprise, and I cannot help saying in conclusion that I think inquiries instituted by your correspondent among some of the leading architects and hospital administration authorities in this country would tend to alter his opinion.

[We invite reports on the durability and length of time in use of jointless flooring in hospitals from our readers. One grave objection to these floors in the past has been the unsightly and unattractive colours they exhibit when laid.—Ed. The Hospital.]

Mr. Alfred Saxon Snell, F.R.I.B.A., writes :

In your issue of November 6, Mr. Milburn, in commending linoleum as a floor covering in wards, shows the methods adopted in three German hospitals for forming the rounded angle between the floor and wall surfaces.

This rounded angle, as he explains, is to avoid "a joint for dirt to get in." He might have added, "and the sharp internal angle which is so difficult to keep clean."

In example No. 1 the floor joint is avoided, it is true, but *three* others are made by the metal strips and one sharp angle. In Nos. 2 and 3, *two* joints and *two* sharp angles.

May I suggest the use of thick linoleum turned up against the wall, with a direct flush joint between it and the plaster? It is quite satisfactory, a I have found by experience. It gives *one* joint only (and that on a vertical surface) and *no* sharp angles.

### GRADUATES' MEDICAL SCHOOLS.

DIARY FOR THE WEEK, NOVEMBER 15 to 20.

### ST. JOHN'S HOSPITAL FOR DISEASES OF THE SKIN, Leicester Square, W.C.

Chesterfield Lectures.—At the Hospital on Thursday evenings at 6 p.m., each lecture followed by clinical demonstrations :—

**November 18, Syphilis**: Treatment (Constitutional and Local in all its forms).

### NATIONAL HOSPITAL FOR THE PARALYSED AND EPILEPTIC, Queen Sq., Bloomsbury, W.C.

**At 3.30 p.m.—November 18,** Dr. Ferrier, **Clinical Lecture.**

**November 19,** Mr. Leslie Paton, **Optic Neuritis.**

### NORTH-EAST LONDON POST-GRADUATE COLLEGE, Prince of Wales's General Hospital, Tottenham, N.

**At 4.30 p.m.—November 16,** Mr. J. H. Evans, **Conditions of the Male Bladder which Produce Bleeding.**

### LONDON SCHOOL OF CLINICAL MEDICINE, Seamen's Hospital, Greenwich, S.E.

**At 2.15 p.m.—November 17,** Dr. F. Taylor.

**November 19,** Dr. Rose Bradford, **Nephritis.**

### MEDICAL GRADUATES' COLLEGE AND POLYCLINIC, 22 Chenies Street, W.C.

**At 4 p.m.—November 15,** Dr. James Galloway, **Skin.**

**At 5.15 p.m.—November 15,** Dr. J. L. Bunch, **Tubercular Diseases of the Skin and their Treatment.**

**At 4 p.m.—November 16,** Dr. C. Theodore Williams, **Medical.**

**At 5.15 p.m.—November 16,** Dr. Wardrop Griffith (Leeds), **On Some Cases and Specimens of Unusual Diseases of the Heart.**

**At 4 p.m.—November 17,** Mr. T. P. Legg, **Surgical.**

**At 5.15 p.m.—November 17,** Mr. Arbuthnot Lane, **Operative Treatment of Fractures.**

**At 4 p.m.—November 18,** Sir Jonathan Hutchinson, **Surgical.**

**At 5.15 p.m.—November 18,** Dr. Francis Warner, **Backward Children and their Training.**

**At 4 p.m.—November 19,** Mr. H. L. Eason, **Eye.**

### THE POST-GRADUATE COLLEGE, West London Hospital, Hammersmith, W.

**At 10 a.m.—November 15** and **18,** Surgical Registrar, **Demonstration.**

**November 19,** Medical Registrar, **Demonstration.**

**At 12 noon.—November 18,** Dr. Bernstein, **Pathological Demonstration.**

**At 12.15 p.m.—November 16,** Dr. Pritchard, **Practical Medicine.**

**November 17** and **20** Dr. Grainger Stewart, **Practical Medicine.**

**At 5 p.m.—November 15,** Mr. Baldwin, **Practical Surgery—II.**

**November 16,** Dr. Reginald Morton, **X-Ray Examination of the Digestive System.**

**November 17,** Dr. Beddard, **Medicine—V.**

**November 18,** Mr. Keetley, **Stiff Joints.**

**November 19,** Dr. Seymour Taylor, **A Common Cold.**

### THE HOSPITAL FOR SICK CHILDREN, Great Ormond Street, W.C.

**At 4 p.m.—November 18,** Dr. Thompson, **Pyelitis in Infancy.**

### ROYAL SOCIETY OF MEDICINE, 20 Hanover Square, W.

**At 8.30 p.m.—November 16, Pathological Section :—**

Dr. M. Haaland : The auto-inoculation and heterologous inoculation of cancer in mice in their practical bearings.

Dr. J. A. Murray : Dissemination of carcinoma by the blood stream in animals.

Dr. B. J. R. Russell : The experimental production of sarcoma during the propagation of a hæmorrhagic adeno-carcinoma of the mouse.

Mr. S. G. Shattock : Oöphorectomy and the uterus in the rabbit.

Mr. Raymond Johnson and Mr. T. W. P. Lawrence : Retro-peritoneal teratoma connected with the spinal canal.

**At 5 p.m.—November 18.—Dermatological Section :—**

Dr. Arthur Whitfield : Localised sclerodermia.

Dr. Graham Little : Lupus erythematosus (acute).

And other cases.

**At 8.30 p.m.—November 19, Electro-Therapeutical Section :—**

Dr. Reginald Morton : The treatment of nævi and other cutaneous lesions by electrolysis, cautery, and refrigeration : with a demonstration on the use of solid carbon dioxide.

**Odontological Section :**—At the next meeting on Monday, November 22 :—

*Paper* : Dr. Eyre and Mr. J. L. Payne : "Some observations on the bacteriology of pyorrhœa alveolaris, and the results of treatment by bacterial vaccines."

*Casual communications* : Mr. C. Schelling : (1) "A case of fracture of the mandible, set with a silver splint made by the casting process." (2) "Two cases of suppuration in the maxilla, in which the Röntgen rays materially helped the diagnosis."

THE HOSPITAL November 13, 1909.

*Name* ..............................

*Address* ..............................

..............................

This Coupon must accompany manuscript or contributions intended for The Hospital.

